# Assessing positive adaptation during a global crisis: The development and validation of the family positive adaptation during COVID-19 scale

**DOI:** 10.3389/fpsyg.2022.886504

**Published:** 2022-09-01

**Authors:** Gillian Shoychet, Dillon T. Browne, Mark Wade, Heather Prime

**Affiliations:** ^1^Department of Psychology, York University, Toronto, ON, Canada; ^2^Department of Psychology, University of Waterloo, Waterloo, ON, Canada; ^3^Applied Psychology and Human Development, University of Toronto, Toronto, ON, Canada

**Keywords:** COVID-19, family functioning, adaptive coping, caregivers, scale validation, measurement invariance

## Abstract

The COVID-19 pandemic has negatively impacted the psychosocial functioning of children and families. It is important to consider adversity in relation to processes of positive adaptation. To date, there are no empirically validated multi-item scales measuring COVID-related positive adaptation within families. The aim of the current study was to develop and validate a new measure: the Family Positive Adaptation during COVID-19 Scale (Family PACS). The sample included 372 female and 158 male caregivers (73% White-European/North American; median 2019 income = $50,000 to $74,999 USD) of children ages 5–18 years old from the United Kingdom (76%), the United States (19%), Canada (4%), and Australia (1%), who completed measures in May 2020. Participants responded to a 14-item survey indexing a range of perceived coping and adaptation behaviors at the beginning of the pandemic. An exploratory factor analysis yielded an optimal one-factor solution comprised of seven items related to family cohesion, flexibility, routines, and meaning-making (loadings from 0.44 to 0.67). Multigroup confirmatory factor analysis demonstrated measurement invariance across female and male caregivers, demonstrating that the factor structure, loadings, and thresholds did not vary by caregiver sex. There was evidence for concurrent validity with significant bivariate correlations between the Family PACS scores and measures of caregiver positive coping, parenting practices, couple satisfaction, and family functioning (correlations from 0.10 to 0.23), but not negatively-valenced constructs. Findings inform our conceptualization of how families have adapted to adverse pandemic-related conditions. Further, we provide preliminary support for the Family PACS as a practical tool for evaluating positive family adaptation during this global crisis, with implications for future widespread crises.

## Introduction

The social disruption caused by COVID-19 has adversely impacted several domains of child, caregiver, and family functioning (Gassman-Pines et al., 2020; Essler et al., 2021). Much of the early research on families focused on pandemic-related disruptions, such as the stressors of homeschooling (Deacon et al., 2021), parent work-life conflict (Wang et al., 2022), loss of income (Wang et al., 2021), and strained parent–child relationships (Cassinat et al., 2021). However, an important part of studying the sequelae of pandemic disruption is to examine processes of resilience—that is, the processes leading to positive adaptation despite exposure to significant threat, adversity, or trauma (Luthar et al., 2000; Masten and Cicchetti, 2016). Resilience is more than merely “bouncing back,” “shaking off,” or “breezing through” life’s challenges; rather, resilience is a dynamic process that affords the potential for personal and relational growth (Walsh, 2016, 2021). Adaptation is one of the many processes that encompass the construct of resilience (Walsh, 1996, 2021; Masten, 2021b). Importantly, such adjustment may not always be positive, as in the case of developing biases in threat perception or aggressive behaviors that promote survival in harmful environments. While these behaviors may be adaptive under settings of risk, they are not necessarily adaptive in settings where risk is low, where they may instead lead to several psychosocial problems (Ellis et al., 2012). In contrast, positive adjustment describes coping-related behaviors that are likely to be adaptive in multiple environments regardless of the level of risk (Ellis et al., 2017). In the current study, we are primarily interested in positive adaptation of families in the context of pandemic stress and disruption.

Early studies on resilience described this construct as situated within the individual—that is, specific traits such as high self-esteem or self-efficacy were examined as precursors to resilience (Rutter, 1987). However, a more nuanced understanding of positive adaptation in response to adversity considers a complex network of interacting systems (individual, relational, and collective; Masten, 2021b). In other words, the capacity for positive adaptation results from ongoing interactions across levels of functioning both within an individual and between the individual and their ever-changing environment (Masten and Cicchetti, 2016; Masten, 2021a). Notably, sources of resilience are themselves dynamic and susceptible to change. The focus of the current paper is the family system as a dynamic entity, subject to processes of risk and resilience during the pandemic (Prime et al., 2020).

Central to a multisystemic framework of positive adaptation is the idea of *family resilience* (Walsh, 1996). Bowen’s (1985) Family Systems Framework views the family as a holistic emotional and functional unit. As family members are interconnected and interdependent, stressors that impact one member have ripple effects on the others (Kerr and Bowen, 1988). Applied to the idea of resilience, family resilience describes the experience of recovery and growth of families following adversity, in the domains of shared belief systems (e.g., meaning-making: when families collectively make sense of stressful and adverse situations), organizational patterns (e.g., flexibility), and communication (e.g., emotional expression; Walsh, 2012, 2016, 2021). The aim of the current paper is to develop and validate a scale of positive family adaptation, which assesses the extent to which caregivers report positive changes to family processes amidst the pandemic.

Exploring patterns of positive family adaptation in response to the pandemic will help articulate processes of risk and resilience in children and families, including how family stressors and positive adaptation interact dynamically to influence family functioning. To date, there are few empirically validated COVID-specific scales measuring family functioning. Some studies include items related to family-specific pandemic stressors (Prime et al., 2021), work-family management strategies (Shockley et al., 2021), and pandemic exposure and impact on family functioning (Kazak et al., 2021). However, no study to date has considered pandemic-related positive adaptation and coping. Moreover, although previous studies included items indexing the positive impact of the pandemic on children (e.g., benefits of home quarantine, parent–child discussions on COVID-19, and family coping strategies), they are not validated scales (Eales et al., 2021; Tang et al., 2021). Thus, an empirically-validated measure of positive family adaptation to pandemic stress and disruption is needed. As the pandemic is likely to have a long-term impact on many children and families, a practical tool for assessing how families are functioning in the months and years ahead is necessary.

There are important sex- and/or gender-based differences that have been demonstrated among parents during the pandemic. Specifically, female caregivers have been reported to experience greater stress and burden during the pandemic than male caregivers (Aguiar et al., 2021; Kerr et al., 2021; Wade et al., 2021). However, it is unclear whether female and male caregivers are positively adapting in different ways in response to pandemic stressors. In general, there is limited research exploring differences between mothers and fathers in processes of resilience. In one study, there were no significant differences in resilience levels (i.e., the capacity to thrive in response to adversity) between mothers and fathers parenting a child with autism spectrum disorder (Bitsika et al., 2013). However, there is some evidence that women and men may have different responses to adversity, which may signal differences in coping or adaptation (Kelly et al., 2008). More research is therefore needed to better understand similarities and differences in the extent or manner in which mothers and fathers cope with pandemic-related stressors.

In giving a questionnaire to individuals who differ on some characteristic (e.g., sex), it is essential to empirically examine whether the construct is meaningful across groups (Millsap, 2011). Invariance testing can be used when creating a new scale to make such a comparison (Bialosiewicz et al., 2013). Measurement invariance is a statistical property of a questionnaire or test that demonstrates that a construct is comparable across groups of people (i.e., multigroup invariance) or across timepoints (i.e., longitudinal invariance; Millsap, 2011; Bialosiewicz et al., 2013; Putnick and Bornstein, 2016; Wang et al., 2018). Measurement invariance is a necessary precondition to testing differences between groups or change over time, particularly because variations in test scores can result for several reasons, many of which are not due to actual differences between groups or timepoints (van de Vijver, 2015). In the case of a measure of family positive adaptation and coping, it is critical to examine whether the measure is invariant across female and male caregivers.

### Current study

The aim of the current study is to develop and validate a caregiver-reported measure of pandemic-related positive adaptation and coping within families, based on research in family resilience (Walsh, 2016; Prime et al., 2020): the Family Positive Adaptation during COVID-19 Scale (Family PACS). Scale development included the following procedures: (1) development of an item pool; (2) administration of items to a large sample; (3) factor analysis; (4) scale formation; and (5) assessment of validity and reliability (Warner, 2013). This study is our first attempt at creating and validating the Family PACS.

First, we assessed the factor structure of the Family PACS using an exploratory factor analysis (EFA). After identifying an optimal structure in the EFA, we examined the factor structure in female and male caregivers, separately, using a confirmatory factor analysis (CFA). Next, we used a multigroup confirmatory factor analysis (MGCFA) to assess measurement invariance across mothers and fathers—specifically, we tested invariance of factor structure (i.e., number of factors and pattern of factor loadings), strength of loadings, and item thresholds (Putnick and Bornstein, 2016). Lastly, we examined reliability (i.e., internal consistency) and concurrent validity in relation to measures of caregiver and family outcomes. We expected positive correlations between the Family PACS and positively-valenced constructs including caregiver positive coping, positive parenting practices, caregiver-partner relationship satisfaction, and family functioning, all in the small range. In addition, we expected negative correlations between the Family PACS and negatively-valenced constructs including anxiety, distress, parenting stress, and negative parenting practices (also in the small range). Validation of the Family PACS will broaden the scope of family-based research by providing a valid and reliable measure to assess positive family processes associated with resilience during and after the COVID-19 pandemic.

## Materials and methods

### Procedure

Data come from the *Child Resilience and Managing Pandemic Emotional Distress in Families Study* (CRAMPED), at the University of Waterloo, with ethics approval from each author’s primary affiliation (i.e., the University of Waterloo, University of Toronto, and York University). The CRAMPED study is a prospective, within-family design. Caregivers with children in the home were recruited *via* Prolific®, a platform that facilitates the research recruitment of a target audience. We aimed to recruit 500 families according to minimum sample considerations for multilevel modelling (Centre for Multilevel Modelling, n.d.). Of the 3,200 participants screened, 626 met inclusion criteria (i.e., ≥18 years of age and have a minimum of two children between 5 and 18 years old), and 549 completed the survey in the allotted time. Supplemental details on sample recruitment can be found elsewhere (Browne et al., 2021; Prime et al., 2021). Although additional waves of data collection occurred in May, July, September, and November 2020, as well as October 2021 and February 2022, only wave one (May 2020) was used in this cross-sectional validation study, as this was the only timepoint in which the Family PACS item pool was administered to participants. A single caregiver read the informed consent and confirmed their participation by selecting a “yes” or “no” response. Additionally, the caregiver responded to survey items about caregiver and child demographics as well as other variables of interest such as COVID-19 stressors, parenting, positive coping, family functioning, and child mental health (among others). Participants were compensated for their time based on the survey length.

### Participants

The sample consisted of 372 female and 158 male caregivers (*N* = 549) who had at least two children ages 5–18 years old (*N* = 1,098; *M_Younger_* = 9.17, *SD* = 3.03; *M_Older_* = 12.24, *SD* = 3.13). Caregivers ranged in age from 24 to 62 years (*M* = 41.33, *SD* = 6.33) and were from the United Kingdom (76%), the United States (19%), Canada (4%), and Australia (1%). Most participants reported White European or White North American racial backgrounds (73%), were married or in common-law relationships (90%), and completed some post-secondary education (64%). Eighty-three percent of the sample did not report any physical or mental condition. Caregivers’ household income in 2019 ranged from < $15,000 to $175,000+ USD (median = $50,000–$74,999 USD; IQR = $25,000–$99,999 USD). In May 2020, 78% of families reported earning less than $6,000 USD a month (median = $2,000–$3,999 USD; IQR = $2,000–$5,999 USD) with most households having 4–5 individuals residing in the home. The present study only uses data from caregiver-reported measures on the younger child (for parenting stress and practices) and themselves.

### Measures

#### Family PACS: Item pool

The primary outcome variable in the current study is pandemic-related positive adaptation within families, measured using the Family PACS. A 14-item pool was developed by the principal investigator of the CRAMPED study (senior author) at the University of Waterloo, and was reviewed by other members of the investigative team (see Prime et al., 2021). Item generation was guided by Walsh’s (2016) Family Resilience Framework. Participants responded using a three-point Likert scale (1 [*Not True*], 2 [*Somewhat True*], and 3 [*Very True*]) to the following prompt: Since the COVID-19 disruption, have any of the following changes occurred in your household? (see Table 1 for a list of items). Items remaining following the validation process comprised the final scale (see Results). Items were summed whereby higher scores represent greater positive family adaptation and coping during COVID-19.

**Table 1:** Item pool.

Item #	Item description
1	Working from home.
**2**	**Engaged in or developed new family activities (e.g., movies, games, outdoor activities, meals, chores, etc.).**
**3**	**Been more physically active.**
4	Used faith/spirituality/religion as a means of coping.
**5**	**Created new family rules.**
6	Children accessed educational materials online.
7	Started homeschooling.
**8**	**Found new meaning in life.**
9	Felt less stressed with regards to work.
**10**	**Prioritized family more than work.**
11	Found more time to rest and be quiet.
**12**	**Reorganized living situation to facilitate working.**
13	Found work as a useful distraction from COVID-19.
**14**	**Other benefits or coping strategies not listed here.**

Items retained in final factor solution are in bold.

#### Validation scales

##### Caregiver outcomes

###### Caregiver anxiety

Caregiver anxiety was measured using the short-form, four-item emotional distress–anxiety measure of the Patient-Reported Outcomes Measurement Information System (PROMIS®; v1.0; PROMIS Health Organization and PROMIS Cooperative Group, 2016). Caregivers responded to the following prompt using a five-point Likert scale ranging from 1 (*Never*) to 5 (*Always*): Please respond to each question or statement by marking one box per question below based on how you have felt in the past 7 days (e.g., “I felt fearful”). Items were summed to create a total score, where higher scores indicate greater caregiver anxiety (ω = 0.91).

###### Caregiver psychological distress

Caregiver stress, anxiety, and depression were measured using the 10-item Kessler Psychological Distress Scale (K10; Kessler et al., 2002). Parents responded to the following prompt: During the past 30 days, e.g., “about how often did you feel nervous?” Parents responded using a five-point Likert scale ranging from 1 (*None of the time*) to 5 (*All of the time*). Items were summed and total scores ranged from 10 to 50. Higher scores represent more psychological distress (ω = 0.93).

###### Parenting stress

Parenting stress was examined using an one-item measure that asked the following question: Over the past 14 days, how stressful were your parenting experiences with [younger child’s name]? (Hartley et al., 2018). Parents responded using a seven-point Likert scale ranging from 1 (*Not at all stressful*) to 7 (*Extremely stressful*).

###### Caregiver positive coping

Positive coping, as operationalized by the Connor-Davidson Resilience Scale (CD-RISC-10; Campbell-Sills and Stein, 2007), refers to one’s capacity to thrive in response to stress and trauma. The CD-RISC-10 includes 10 items asking caregivers to what extent a given statement applied to them (e.g., “I am able to adapt when changes occur”). Each item is rated on a 5-point Likert scale, ranging from 0 (*Not at all true*) to 4 (*True nearly all the time*). A total score ranging from 0 to 40 was calculated by summing all the items. Higher scores indicate greater levels of positive coping (ω = 0.92).

##### Family outcomes

###### Parenting practices

Parenting practices were measured using the 10-item revised version of the Parenting Practices Scale from the 2014 Ontario Child Health Study (Boyle et al., 2019). Caregivers responded to the following prompt: Please read each statement below and mark the circle that most closely describes the way you have acted toward (younger child’s name) in the past month. The scale includes five positive (e.g., “I enjoy doing things with [younger child’s name]”) and five negative (e.g., “I get angry and yell at [younger child’s name]”) items measuring parenting practices. The scale ranged from 1 (*Never*) to 5 (*Always*) in which scores were summed to form a five-item positive and five-item negative subscale. Higher scores on the positive and negative subscales represent greater positive (ω = 0.85) and negative (ω = 0.80) parenting practices, respectively.

###### Caregiver-partner relationship satisfaction

Relationship satisfaction was assessed using the four-item version of the Couples Satisfaction Index (Funk and Rogge, 2007). Items are rated on a six- or seven-point Likert scale and measure happiness, warmth, reward, and satisfaction within the couple relationship (e.g., “I have a warm and comfortable relationship with my partner”). A composite score was calculated in which higher scores denote greater relationship satisfaction (ω = 0.94).

###### Family functioning

Family functioning was measured using the validated shortened version of the General Functioning subscale (Boterhoven de Haan et al., 2015) of the Family Assessment Device (FAD; Epstein et al., 1983). Participants were asked the following: Please rate your level of agreement with each of these statements based on your family. The scale is comprised of the six positive items from the General Functioning (GF6+) subscale (e.g., “We can express feelings to each other”). Items were scored using a four-point Likert scale, ranging from 1 (*Strongly Agree*) to 4 (*Strongly Disagree*). The GF6+ total score was calculated using the mean of all six items, where higher scores represent more family dysfunction (ω = 0.87).

### Data analytic plan

We used Mplus Version 8.7 (2012–2021) to run the EFA, CFA, and MGCFA. SPSS Statistics (Version 28.0) was used to compute descriptive statistics, internal consistency, and concurrent validity.

#### Exploratory factor analysis

First, we examined the distributions of each item to assess skewness (i.e., if ≥90% of participants endorse one out of the three scale points). EFA aims to assess the number and nature of latent variables (*common factors*) that explain the variance and covariance among manifest variables (*indicators*; Brown, 2015). EFA is used during the early stages of scale validation when there are no pre-established hypotheses regarding the relationships between the latent and manifest variables (i.e., when the *factor loadings* are unknown; Brown, 2015). We examined a scree plot to determine the number of potential factors to explore within an EFA (eigenvalues >1). Based on this, we subjected measured variables to an EFA, with consideration of one to four factors, using a geomin (oblique) rotation and the default weighted least squares estimator for categorical/ordinal indicators. The optimal factor solution was determined by considering both statistical and conceptual fit, including model fit, factor loadings ≥0.40, theoretical plausibility of the factors, and retainment of measured variable items. Individual factors were considered if they had four or more indicators (Fabrigar et al., 1999; Watkins, 2018). Model fit cut-offs included a non-significant chi-square value, the comparative fit index (CFI) ≥ 0.95, and the root mean square error of approximation (RMSEA) ≤ 0.06. One limitation of using the chi-square test of model fit is that large sample sizes are more likely to produce significant chi-square values regardless of model fit (Byrne, 2011; Kite and Whitley, 2018). As such, if the CFI/RMSEA met indicated acceptable fit, then a significant chi-square value was ignored in determining model fit (Kite and Whitley, 2018).

#### Measurement invariance

Once we established the optimal factor structure, we ran a CFA to examine the factor structure in female and male caregivers, separately, and to address misspecified parameters. Next, using the Mplus shortcut, we examined measurement invariance through a MGCFA, with caregiver sex (female vs. male) as the grouping variable. MGCFA enables researchers to test for measurement invariance by assessing the degree of model homogeneity between groups (Wang et al., 2018). Each level of measurement invariance is tested using a ladder-like approach, starting with the least restrictive to most restrictive hypotheses (Bialosiewicz et al., 2013; Putnick and Bornstein, 2016).

The first and most basic type of measurement invariance is *configural invariance*, which requires that the same factor structure (i.e., number of factors and pattern of factor loadings) is supported in both groups (Putnick and Bornstein, 2016; Wang et al., 2018). The configural model is the baseline model without any constraints. Although the factor structure must be identical, the actual strength of each factor loading can vary across groups (Bialosiewicz et al., 2013; Wang et al., 2018). The next step typically involves establishing *metric invariance*, which requires that items load onto latent factors similarly across groups (i.e., the strength of the factor loadings must be the same; Putnick and Bornstein, 2016). However, metric invariance testing is not computed separately when using the weighted least squares mean and variance adjusted estimator for ordinal indicators in Mplus (WLSMV; Muthén and Muthén, 1998–2017; Putnick and Bornstein, 2016). Thus, metric invariance was tested simultaneously with *scalar invariance*, which requires both factor loadings and thresholds to be equal across groups (Putnick and Bornstein, 2016; Wang et al., 2018). Metric and scalar invariance refer to the “scalar model” hereafter.

The MGCFA computes chi-square, CFI, and RMSEA values for each of the configural and scalar models, and provides a statistical comparison of model fit (e.g., scalar against configural). A non-significant chi-square difference test indicates that the scalar model is not significantly worse than the configural model, establishing scalar invariance (Putnick and Bornstein, 2016). However, since chi-square tests are sensitive to sample size, additional cut-off criteria were included to establish model fit (i.e., ΔCFI ≤ −0.01 and ΔRMSEA ≤ 0.01, both of which refer to magnitude of change; Cheung and Rensvold, 2002; Chen, 2007; Putnick and Bornstein, 2016).

#### Scale formation and concurrent validity

After establishing measurement invariance, items were summed to create a total score based on the number of established factors. We used McDonald’s (1999) coefficient omega to examine internal consistency as it is a robust parameter of reliability that is not constrained by the stricter assumptions required to use coefficient alpha or the number of scale items (Dunn et al., 2014; Tavakol, 2018). We used Spearman’s rho to test for concurrent validity between the Family PACS and caregiver and family variables.

## Results

### Exploratory factor analysis

**Figure 1:**
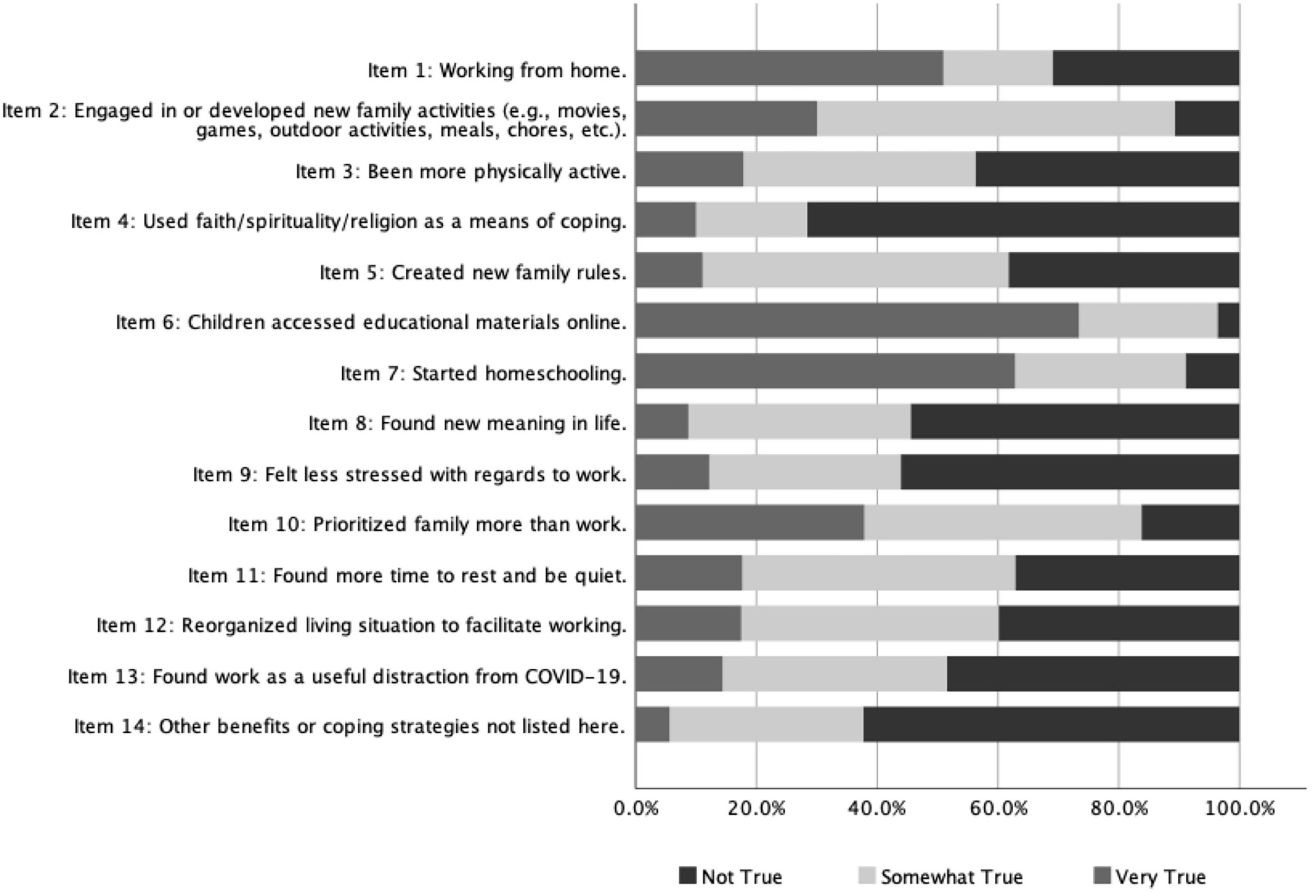
Frequency distribution of responses from the Family PACS item pool. Family PACS, Family Positive Adaptation during COVID-19 scale.

We retained all 14 items from the Family PACS item pool following an assessment of the frequency distributions of responses (see Figure 1). A scree plot of eigenvalues indicated a potential four-factor structure. We subjected the 14 measured variables to an EFA, with consideration of one to four factors. In the four-factor model, one factor included only two indicators with factor loadings ≥0.40 (item 6 and 7, related to schooling/online learning). These items were not conceptually meaningful as they did not inherently signal coping vs. stress as it relates to schooling. We next examined one-to-three factor solutions with these items removed. There was support for a conceptually-meaningful three-factor solution reflecting flexibility (items 1, 12, and 13), meaning-making (items 8, 9, 10, and 11), and routines/rituals (items 2, 3, 5, and 8), with good model fit (CFI = 0.978; RMSEA = 0.052). However, as one factor included only three indicators with factor loadings ≥0.40, and there was cross-loading of item 8 across two factors, we did not explore this factor structure further. Finally, in comparing one- and two-factor solutions, a conceptually-meaningful one-factor solution accounted for approximately 22.8% of total variance, with optimized item retainment with factor loadings ≥0.40, and good model fit (CFI = 0.966; RMSEA = 0.051). This one-factor model was selected as the final factor structure. The seven items and factor loadings (ranging from 0.44 to 0.67) for the one-factor solution can be seen in Table 2.

**Table 2:** Exploratory Factor Analysis One-Factor Solution (Geomin Rotated Loadings).

Item #	Item description	1
2	Engaged in or developed new family activities	0.670^*^
3	Been more physically active.	0.579^*^
5	Created new family rules.	0.440^*^
8	Found new meaning in life.	0.598^*^
10	Prioritized family more than work.	0.440^*^
12	Reorganized living situation to facilitate working.	0.504^*^
14	Other benefits or coping strategies not listed here.	0.515^*^

* *p* < 0.05.

### Measurement invariance

Confirmatory factor analysis models were conducted for each caregiver sex grouping, separately, with support for the factor structure in both female and male caregivers. Factor loadings in male (*n* = 158; loadings from 0.43 to 0.73) and female caregivers (*n* = 372; loadings from 0.42 to 0.67), as well as CFI/RMSEA, were acceptable for both groups (see Table 3). There were no misspecified parameters in either group.

**Table 3:** Structural and measurement invariance model fit indices.

	χ^2^ (df)	CFI	RMSEA	ΔCFI	ΔRMSEA	Δχ^2^(df)	Decision
One-factor EFA (*N* = 549)	33.833(14)^*^	0.966	0.051	–	–	–	–
	MEASUREMENT INVARIANCE: ONE-FACTOR MODEL						
Female (*n* = 372)	26.623(14)^*^	0.964	0.049	–	–	–	–
Male (*n* = 158)	19.338(14)	0.979	0.049	–	–	–	–
Configural invariance (*n* = 530)	46.141(28)^*^	0.970	0.049	–	–	–	–
Scalar invariance^a^ (*n* = 530)	62.524(40)^*^	0.963	0.046	−0.007	−0.003	17.890(12)	Accept

CFI, comparative fit index; RMSEA, root mean square error of approximation; and *χ*^2^, chi-square. χ^2^, CFI, and RMSEA are absolute model fit indices, whereas ΔCFI, ΔRMSEA, and Δχ^2^ compare two nested models.

a Compared to Configural.

* *p* < 0.05.

#### Configural model

The baseline model without any constraints had good model fit. Although the chi-square test of model fit was significant, χ^2^ (28, *n* = 530) = 46.14, *p* = 0.017, the cut-offs were met for the two test indices not affected by sample size (CFI = 0.970; RMSEA = 0.049; Kite and Whitley, 2018). Thus, the one-factor structure demonstrated configural invariance across female and male caregiver groups (Cheung and Rensvold, 2002; Chen, 2007; Putnick and Bornstein, 2016).

#### Scalar model

The scalar model had good model fit; despite the significant chi-square test, χ^2^(40, *n* = 530) = 62.52, *p* = 0.013, both the CFI and RMSEA values were within the appropriate range for determining good model fit (CFI = 0.963; RMSEA = 0.046). Next, chi-square difference testing revealed that the scalar model did not worsen model fit compared to the configural model, Δχ^2^ (12, *n =* 530) = 17.89, *p* = 0.119. This conclusion was supported by additional fit indices (ΔCFI = −0.007 and ΔRMSEA = −0.003; Cheung and Rensvold, 2002; Chen, 2007; Putnick and Bornstein, 2016). Thus, there is evidence for configural (i.e., factor structure), metric (i.e., factor loadings), and scalar (i.e., item thresholds) invariance in the one-factor model across female and male caregivers.

### Scale formation and concurrent validity

A total score for the seven-item scale was computed by summing all items. McDonald’s Omega for the total sample was ω = 0.65 (ω_female_ = 0.64; ω_male_ = 0.70). Spearman’s rho correlations were used to examine concurrent validity between the total score and other validated caregiver and family measures expected to relate to COVID-19 family coping and adaptation (see Table 4). Only the positively-valenced constructs (caregiver positive coping, positive parenting practices, couple satisfaction, and family functioning) were significantly correlated with the Family PACS score, with associations in the small range. Negatively-valenced constructs (caregiver anxiety, caregiver psychological distress, parenting stress, and negative parenting practices) were not significantly correlated with the Family PACS score.

**Table 4:** Correlations between the Family PACS and caregiver and family outcomes.

	Family PACS
	Caregiver Outcomes
Anxiety (*n* = 545)	0.057
Psychological distress (*n* = 546)	−0.025
Parenting stress (*n* = 547)	0.020
Positive coping (*n* = 547)	0.232^***^
	Family Outcomes
Positive parenting practices (*n* = 547)	0.218^***^
Negative parenting practices (*n* = 547)	0.029
Couple satisfaction^a^ (*n* = 507)	0.103^*^
Family functioning (higher is worse; *n* = 549)	−0.132^**^

*n* = sample size included in analysis. Family PACS = Family Positive Adaptation during COVID-19 Scale.

a Valid missingness due to skips (no partner).

*** *p* < 0.001;

** *p* < 0.01;

* *p* < 0.05.

## Discussion

The widespread social disruption caused by the COVID-19 pandemic has negatively impacted the functioning and well-being of many families around the world. Families with school-aged children have experienced financial burden (Low and Mounts, 2022), food insecurity (Steimle et al., 2021), school closures (Almeida et al., 2021), and lack of child support (Poulain et al., 2021), with associated strain on the functioning of the family system (Prime et al., 2020). Notwithstanding its negative consequences, many families have been able to mobilize social, interpersonal, and familial resources to adapt to these disruptions and cope with the stress that accompanies them. Yet measures that capture this family-based resilience have been largely overlooked. A focus on coping and positive adaptation is crucial not only to understand how families in the current pandemic will recover from the disruption it has created, but also for future disaster mitigation. Thus, development of measures that capture the ability of families to overcome, endure, or otherwise cope with significant social disruption caused by the pandemic are essential to current recovery efforts and planning for future crises. The current study provides one of the first attempts to validate such a measure, the Family PACS, in a relatively large multinational sample of female and male caregivers.

Our findings provide initial validation of a very brief, easily implemented scale indexing several constructs important for family resilience in the face of adversity (Walsh, 1996, 2016). The seven-item Family PACS maps onto themes of meaning-making (e.g., “Found new meaning in life”), connectedness (e.g., “Engaged in or developed new family activities”), and social and economic resources (e.g., “Reorganized living situation to facilitate working”). Items within the Family PACS align with emerging international evidence during the pandemic. Specifically, a comprehensive literature review found that constructs of adaptability, family cohesion, re-creating routine, and flexibility were all important contributors to enhancing family resilience during the pandemic (Gayatri and Irawaty, 2022). Our findings build on this important work by putting forth a valid and reliable tool for measuring key constructs of family resilience aligned with emerging literature. This will allow for consistency across studies in measuring pandemic-specific family positive adaptation.

Importantly, the one-factor structure of the Family PACS demonstrated strong measurement invariance. That is, the factor structure (i.e., number of factors and pattern of factor loadings), strength of factor loadings, and item thresholds were indistinguishable by caregiver sex. Strong measurement invariance is a necessary condition to testing differences between groups to ensure that significant differences in scores are attributed to actual population differences and not a result of measurement bias (Bialosiewicz et al., 2013; Wang et al., 2018). Thus, our findings will enable researchers to explore whether there are sex differences in experiences of family coping and positive adaptation during the pandemic. This is important as previous research has found differences in coping styles and stress responses between females and males (e.g., Kelly et al., 2008; Wade et al., 2021).

The Family PACS had acceptable concurrent validity, with positive correlations in the small range with positively-valenced items. This demonstrates that the Family PACS, which asks questions specific to pandemic-related processes, is related to constructs of other, more stable, positive family processes including caregiver positive coping (e.g., “I am able to adapt when changes occur”), positive parenting (e.g., “I enjoy doing things with [younger child’s name]”), couples’ satisfaction (e.g., “I have a warm and comfortable relationship with my partner”), and family functioning (e.g., “We can express feelings to each other”). It was initially surprising that the Family PACS was not significantly correlated with any of the negatively-valenced scales since past research has demonstrated that dimensions of stress, anxiety, and distress correlate with coping behaviors (Compas et al., 2019). One possible explanation is that, very early in the pandemic (May 2020), processes of positive adaptation and negative caregiver and family processes were operating independently. That is, positive adaptation may not simply be the inverse of maladjustment. As risk and resilience are dynamic constructs (Masten, 2021a), the associations between the Family PACS and negatively-valenced scales of interest may be more strongly related at later points in the pandemic, or when assessing predictive validity. Despite this, it is promising that our measure of positive adaptation is related to positive family processes.

Notably, there was initial support for a three-factor structure with acceptable factor loadings and conceptually meaningful themes (i.e., flexibility, meaning-making, and routines/rituals), which was not pursued due to few indicators on factors. However, this provides some support for a multidimensional scale, which is corroborated by the acceptable, but not optimal, internal consistency of the Family PACS. As such, next steps may include item development to increase the number of measured variables aligned with the identified factors, with the goal of creating a multidimensional scale. Relatedly, there are other relevant items that may strengthen the Family PACS. For example, items related to having a positive outlook (hope, confidence, and “can-do spirit”) and open emotional sharing (expression of negative feelings, shared gratitude, humor; Walsh, 2016, 2021) may provide additional insight into family adaptation during this time. Further item development of the scale may lead to improvements in both validity and reliability. At present, the current brief scale may be useful in capturing processes related to positive family adaptation during the pandemic, with potential to facilitate research in the areas of family risk and resilience. As the Family PACS asks about experiences specific to the pandemic, adjustments and further evaluation will be necessary if it is implemented as a measure of family positive adaptation during other adverse circumstances.

### Limitations

Researchers who use this scale should be cognizant of the limitations inherent to the sampling approach. Specifically, most of the sample was White European or North American, married, well-educated, and they had on average middle to high income. These specific demographic groups typically experience lower risk than those with low income, limited education, single-parenthood, and/or individuals from racialized groups (Radey et al., 2021; Lewis et al., 2022). This has potential consequences for the cultural sensitivity of the measure and applicability to diverse groups. In developing the measure, our goal was to create broadly applicable items. However, some of the Family PACS items may be biased towards certain groups, such as middle to high income families (e.g., “Prioritized family more than work”). Moreover, some items relevant to specific groups may have been omitted. For example, drawing on social support and/or parenting support may be particularly critical to positive adaptation for some caregivers during the pandemic, such as single parents, essential workers, and healthcare workers (Lee et al., 2009; Taylor and Conger, 2017; Stevenson et al., 2021). Consequently, we are not able to speak to whether the measure is valid beyond the sample it was validated on, which is a largely homogenous group. Taken together, the Family PACS will need to be validated and potentially adapted when applied to new samples.

Regarding the operationalization of positive adaptation, due to the general wording of items, participants may have interpretated the items in different ways. For example, although we intended the item “Found new meaning in life” to be understood as a collective/family concept based on Walsh’s (2016) view of family resilience, some participants may have interpreted the item as an individual concept. Similarly, participants may have interpreted meaning-making as a set of behaviors, beliefs, or both. As we did not provide specific definitions of constructs to participants, their responses are solely based on their individual interpretations. Accordingly, this is a risk for several items and warrants consideration in interpreting scale meaning.

Another consideration in scale development is the timing of data collection (May 2020), a mere two months following the declaration of COVID-19 as a pandemic. It is likely that processes of risk and resilience have evolved over the years following. Indeed, some processes of adaptation may be more salient during times of acute adversity and others during chronic adversity. For example, during the 1980s economic recession, families engaged in various coping strategies to deal with their imminent financial difficulties, such as having open dialog with their children about practical strategies they could employ (e.g., reducing expenses; Lucas and Buzzanell, 2012). Although these forms of communication fostered short-term resilience, parents also used their hardship to teach their children valuable lessons that would foster character development and long-term resilience (Lucas and Buzzanell, 2012). As chronicity is important to consider when examining prolonged adversity at a single time point, future work should account for how processes of resilience may change over time.

Some final sample considerations relate to the study design. Specifically, all caregivers were required to have a minimum of two children to participate, as the overarching design of the CRAMPED project was a within-family (sibling-comparison) design. Therefore, findings may not be generalizable to single-child households. Additionally, our study comprised unbalanced samples between males and females. This is not inherently problematic as it does not introduce statistical bias; however, the estimates specific to the smaller male group may be less precise than those specific to the female group (Chen, 2007). Finally, the aim of the CRAMPED study was to examine the social and economic disruption that has impacted so many families during the pandemic, rather than medical conditions or diseases (including COVID-19 specifically). As such, the Family PACS is a measure of positive adaptation in response to psychosocial stressors and not biomedical stressors, and therefore should be interpreted as such.

### Conclusion

Although the pandemic has had significant negative effects on the family system, there are many families who have been able to positively adapt to these adverse circumstances. Findings from the current study provide initial evidence for a useful short-item scale assessing positive adaptation in families. Gaining a better understanding of the factors that promote processes of resilience in children, caregivers, and families during COVID-19 aid current pandemic recovery efforts, and inform future attempts to mitigate international crises.

## Data availability statement

The raw data supporting the conclusions of this article will be made available by the authors, without undue reservation.

## Ethics statement

The studies involving human participants were reviewed and approved by the University of Waterloo, University of Toronto, and York University. The patients/participants provided their written informed consent to participate in this study.

## Author contributions

GS: conceptualization, analysis, and writing. DB: conceptualization, methodology, data collection, reviewing, and editing. MW: reviewing and editing. HP: analysis, writing, reviewing, and editing. All authors contributed to the article and approved the submitted version.

## Funding

This work was funded by Tri-agency/Canadian Government sponsor; Canadian Government Agency: SSHRC—Social Sciences and Humanities Research Council; Program Name: Canada Research Chair; Work-order/award number: # 950-232347 (DB); and by Sciences and Humanities Research Council Insight Grant; Work-order/award number: 435-2019-1052 (HP).

## Conflict of interest

The authors declare that the research was conducted in the absence of any commercial or financial relationships that could be construed as a potential conflict of interest.

## Publisher’s note

All claims expressed in this article are solely those of the authors and do not necessarily represent those of their affiliated organizations, or those of the publisher, the editors and the reviewers. Any product that may be evaluated in this article, or claim that may be made by its manufacturer, is not guaranteed or endorsed by the publisher.
